# 2629. Estimation of Hospitalization and Mortality Burden of RSV in Adults in Spain between 2015–2019

**DOI:** 10.1093/ofid/ofad500.2242

**Published:** 2023-11-27

**Authors:** Mariana Haeberer, Antoni Torres, Robin Bruyndonckx, Aleksandra Polkowska-Kramek, Charles Nuttens, Francesca Lemme, Thao Mai Phuong Tran, Worku Biyadgie Ewnetu, Caihua Liang, Cristina Mendez Diez, Bradford D Gessner, Elizabeth Begier

**Affiliations:** Pfizer, Madrid, Madrid, Spain; Clínic Barcelona Hospital, Spain, Catalonia, Cantabria, Spain; P95, Leuven, Brabant Wallon, Belgium; P95, Leuven, Brabant Wallon, Belgium; Pfizer Inc, Paris, Ile-de- France, France; P95, Leuven, Brabant Wallon, Belgium; P95, Leuven, Brabant Wallon, Belgium; P95, Leuven, Brabant Wallon, Belgium; Pfizer Inc, Paris, Ile-de- France, France; Pfizer, Madrid, Madrid, Spain; Pfizer Biopharma Group, Collegeville, Pennsylvania; Pfizer Vaccines, Dublin, Dublin, Ireland

## Abstract

**Background:**

Respiratory syncytial virus (RSV) can cause serious respiratory illness and complications of cardiovascular diseases, especially in older adults. RSV burden in adults is underestimated, due to nonspecific symptoms and infrequent testing. We retrospectively estimated the RSV-attributable incidence of hospitalizations and deaths applying two model-based approaches.

**Methods:**

Analyzing nationwide data from hospital discharges (2016–2019) and mortality (2015–2019), we estimated the RSV-attributable incidence rate of cardiorespiratory hospitalizations and deaths stratified by age and disease subgroups (5 respiratory;5 circulatory). We used a quasi-Poisson regression model, accounting for seasonal fluctuations and RSV and influenza activity. A hierarchical Bayesian model was run as a sensitivity analysis.

**Results:**

RSV-attributable incidence rates of hospitalizations increased with age. The highest incidence was observed in those aged ≥80 years admitted for respiratory diseases (representing 9-10% of all respiratory hospitalizations, vs 3% in 18-49 age group). Considering secondary outcomes, rates were higher for chronic heart failure and arrythmias among the oldest age group, while among younger age groups they were higher for chronic obstructive pulmonary disease (COPD) exacerbation (50-59 and 60-79 years) and pneumonia/influenza/bronchitis (18-49 years)(Table). RSV-attributable mortality rates were higher for circulatory compared with respiratory causes in all age groups, especially due to chronic heart failure exacerbation (age ≥80 years) and acute coronary event/myocardial infarction (age 60-79 years). Bayesian model had less optimal model fit and tended to obtain lower estimates for hospitalizations that were substantially lower than prospective estimates from other high-income countries.

**Figure:**
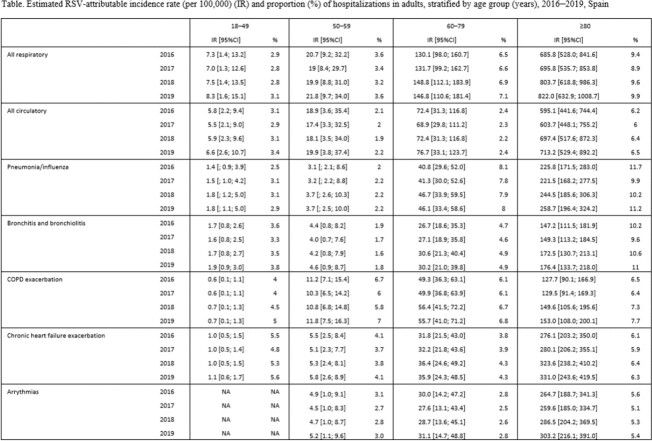

**Conclusion:**

These model-based estimates are comparable to those from observational studies and suggest that RSV causes a considerable burden of hospitalizations in adults. Like other respiratory viruses (e.g, influenza and SARS-CoV-2), RSV contributes to both respiratory and circulatory hospitalizations. This suggest that efficacious vaccines could have a high public health impact.

**Disclosures:**

**Mariana Haeberer, PhD**, Pfizer: Employee **Robin Bruyndonckx, n/a**, P95: RB is an employee of P95, paid by Pfizer to conduct the study **Aleksandra Polkowska-Kramek, n/a**, P95: APK is an employee of P95, paid by Pfizer to conduct the study **Charles Nuttens, n/a**, Pfizer: CN is an employee of Pfizer, the sponsor of this study **Thao Mai Phuong Tran, n/a**, P95: TMPT is an employee of P95, paid by Pfizer to conduct the study **Worku Biyadgie Ewnetu, n/a**, P95: WBE is an employee of P95, paid by Pfizer to conduct the study **Caihua Liang, MD, PhD**, Pfizer: CL is an employee of Pfizer, the sponsor of this study **Cristina Mendez Diez, PhD**, Pfizer: Employee **Bradford D. Gessner, M.D., M.P.H.**, Pfizer: I am an employee of Pfizer|Pfizer: Stocks/Bonds **Elizabeth Begier, M.D., M.P.H.**, Pfizer: EB is an employee of Pfizer, the sponsor of this study|Pfizer: Stocks/Bonds

